# Advances in the treatment of lower-extremity ischemia-reperfusion injury

**DOI:** 10.3389/fphar.2025.1576091

**Published:** 2025-05-23

**Authors:** Jin Peng, Tang Deng, Xunkai Wang, Jinxi Liang, Jiangpeng Wu, Binglong Li, Jun Lv, Shenmei Wu, Shijie Zhong, Chen Yao, Guiyun Jin

**Affiliations:** ^1^ Department of Interventional Radiology and Vascular Surgery, The First Affiliated Hospital, Hainan Medical University, Haikou, China; ^2^ Department of Emergency, The First Affiliated Hospital, Hainan Medical University, Haikou, China; ^3^ Key Laboratory of Emergency and Trauma Ministry of Education, College of Emergency and Trauma, Hainan Medical University, Haikou, China; ^4^ Division of Vascular Surgery, The First Affiliated Hospital, Sun Yat-sen University, Guangzhou, China; ^5^ Department of Ophthalmology, The First Affiliated Hospital of Guangxi Medical University, Nanning, China

**Keywords:** ischemia-reperfusion, lower limbs, antioxidant, oxidative stress, treatment

## Abstract

Lower-limb ischemia–reperfusion injury (LL-IRI) is a frequent and serious complication following reperfusion therapy for lower-limb arterial occlusion. It can be caused by trauma, arterial stenosis, thrombotic occlusion, and atherosclerosis. As a prevalent peripheral vascular disease, LL-IRI results in local tissue damage and triggers systemic inflammatory responses that can lead to multiple-organ dysfunction syndrome, as well as multiorgan failure and death in severe cases. Despite its clinical significance, the mechanisms underlying IRI remain poorly understood, and no specific targeted drugs or effective emergency interventions are currently available. Therefore, this review provides a comprehensive analysis of the domestic and international literature published over the past decade regarding the disease definition, pathogenesis, and therapeutic advances in LL-IRI. It summarizes current pharmacological and non-pharmacological interventions, including antioxidant stress management, anti-inflammatory approaches, and oxidative stress reduction strategies. This review aims to advance the exploration of LL-IRI pathogenesis and proposes novel therapeutic perspectives for innovative drug development.

## Introduction

Ischemia-reperfusion syndrome was described by Haimovici in 1960 as a serious complication following acute ischemic surgery (Magnoni et al., 1996). Ischemia–reperfusion injury (IRI) is a syndrome in which tissues, after experiencing a period of ischemia, experience exacerbated damage and organ dysfunction upon the restoration of blood flow (Cai J. et al., 2022). It involves a systemic immune response triggered by the release of pro-inflammatory mediators and reactive oxygen species (ROS) during the reperfusion of ischemic organs and/or tissues. IRI often surpasses the damage caused by ischemia alone (Cearra et al., 2021), and it has been extensively studied across various tissues and organs.

Lower-limb IRI (LL-IRI) is among the most common peripheral vascular diseases, typically induced by chronic arterial narrowing (*e.g.*, arterial stenosis, thrombotic occlusion), trauma, atherosclerosis, or surgical procedures using tourniquets to create bloodless surgical fields. Acute embolism, such as that resulting from atrial fibrillation, leading to arterial obstruction in the lower limbs can also lead to tissue hypoxia and metabolic imbalance, potentially triggering IRI (Eltzschig and Eckle, 2011). In severe cases, LL-IRI can cause damage to distal organs (*e.g.*, lungs, kidneys, liver), progressing to multiple-organ dysfunction syndrome (Cai et al., 2018).

Current research into IRI treatments has extensively focused on cardiac and cerebral applications, with fewer studies devoted to LL-IRI treatment. This review discusses recent studies on antioxidant therapies for LL-IRI, covering multiple aspects such as anti-oxidation, mitochondrial injury mitigation, anti-inflammation, endothelial cell protection, angiogenesis promotion, and anti-apoptosis strategies.

## Mechanisms of LL-IRI

Peripheral artery disease (PAD) is a major public health burden characterized by circulatory disorders in the lower limbs, which restrict their mobility. Atherosclerotic lesions narrow the arteries, leading to ischemia (Liang et al., 2021). Skeletal muscle, the predominant tissue in the limbs, is highly susceptible to ischemia. Recent physiological and anatomical research indicates that 3 h of ischemia can induce severe skeletal muscle injury (de Carvalho et al., 2023). PAD is manifested by reduced oxygenation of the lower limbs, which modern medical interventions address by restoring blood flow to the affected areas. Whereas limb revascularization effectively alleviates symptoms of ischemia, it also causes several adverse effects, one of the most severe being reperfusion injury (Saran and Malarkey, 2019). Mild LL-IRI can present as slight swelling, pain, and small skin blisters in the lower limbs. In severe cases, muscle and nerve tissues confined within the fascial compartments of the bones can experience secondary ischemia attributable to increased pressure, leading to diminished or lost distal limb sensation, restricted toe or ankle joint function, severe lower-limb swelling, pain, and potentially extensive skin blistering or necrosis. During the ischemia-reperfusion process, toxic substances such as oxygen free radicals, inflammatory factors, acidic metabolites, and various ions re-enter the bloodstream, damaging multiple organs and impairing their function. In severe instances, this can lead to multiorgan failure and life-threatening conditions (Zhang et al., 2024).

**FIGURE 1:**
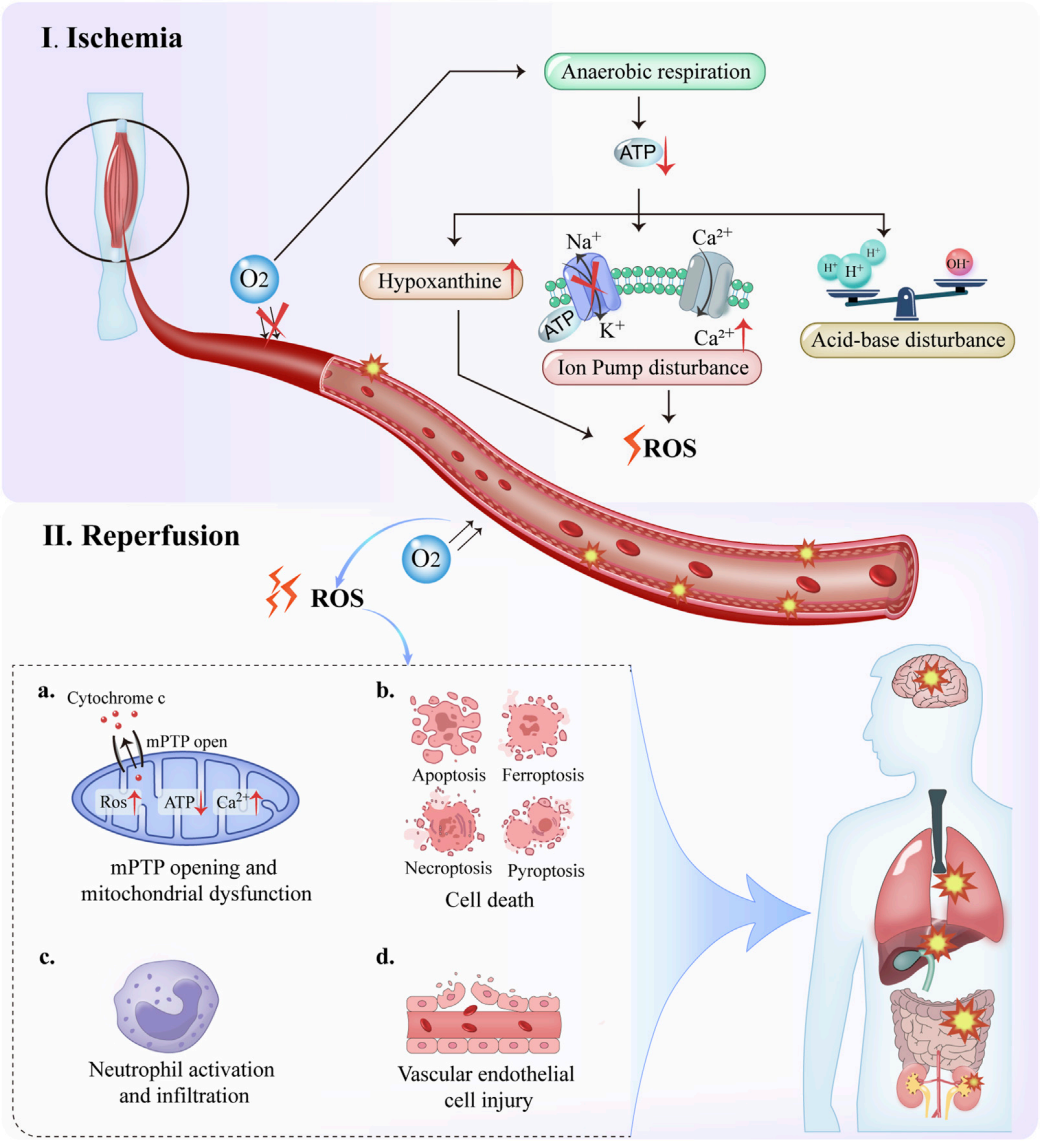
Mechanism of lower-limb ischemia-reperfusion injury. Lower-limb injury can be divided into two stages: ischemia and reperfusion.

LL-IRI can be divided into two distinct phases: ischemia and reperfusion. During ischemia, arterial flow obstruction leads to hypoxia and inadequate perfusion, impairing the normal function of the mitochondrial electron transport chain (Şengel et al., 2023). Following reperfusion, blood flow to the ischemic tissues is restored as red blood cells deliver oxygen. However, this process also leads to increased ROS generation because of the reduced antioxidant concentrations in ischemic cells. Elevated ROS levels can induce oxidative stress, resulting in endothelial dysfunction, DNA damage, and local inflammatory responses (Figure 1) (Wu et al., 2018). LL-IRI exacerbates rhabdomyolysis through several interconnected mechanisms: calcium overload activates proteases, leading to myofibrillar protein degradation; ROS induce sarcolemmal rupture via lipid peroxidation; and permeability transition pore (PTP)-mediated ATP depletion impairs cellular energy homeostasis. These pathways collectively promote myoglobin release and systemic inflammatory responses (Cabral et al., 2020).

Ischemic stage: O_2_ content in the body is reduced. Anaerobic respiration cannot be performed, and only anaerobic respiration is possible, leading to reduced ATP levels and the following three conditions: hypoxanthine content is greatly increased; ion pumps are disturbed, and acidosis occurs because of anaerobic respiration and the increase of hydrogen ions in the body, causing acid-base imbalance throughout the body.

Reperfusion stage: With the recovery of O_2_ content *in vivo*, reactive ROS levels significantly increase, resulting in the four conditions: mitochondrial permeability transition pore (mPTP) opening and mitochondrial dysfunction; cell death (four main modes: apoptosis, pyroptosis, necroptosis, and ferroptosis); vascular endothelial cell injury; and neutrophil activation and infiltration.

These two process es cause damage to other tissues throughout the body.

**FIGURE 2:**
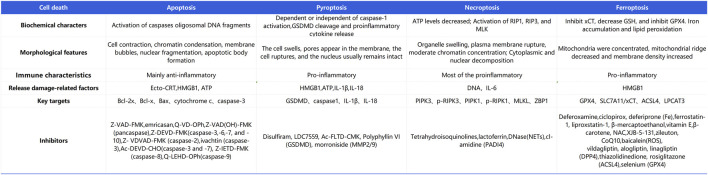
Differences in the major modes of cell death in lower-limb ischemia-reperfusion injury.

During LL-IRI, multiple modes of cell death are concurrently activated, including apoptosis, pyroptosis, ferroptosis, and necroptosis. Cell death can be categorized into two types: regulated and accidental cell death (Liang et al., 2021). Regulated cell death involves signaling cascades mediated by effector molecules and exhibits distinctive biochemical and morphological features (Tong et al., 2022). Conversely, accidental cell death is triggered by unexpected injury stimuli that exceed the cell’s regulatory capacity, leading to various forms of cell demise such as necroptosis and pyroptosis. Each form is characterized by distinct morphological and biochemical traits, as depicted in Figure 2.

**FIGURE 3:**
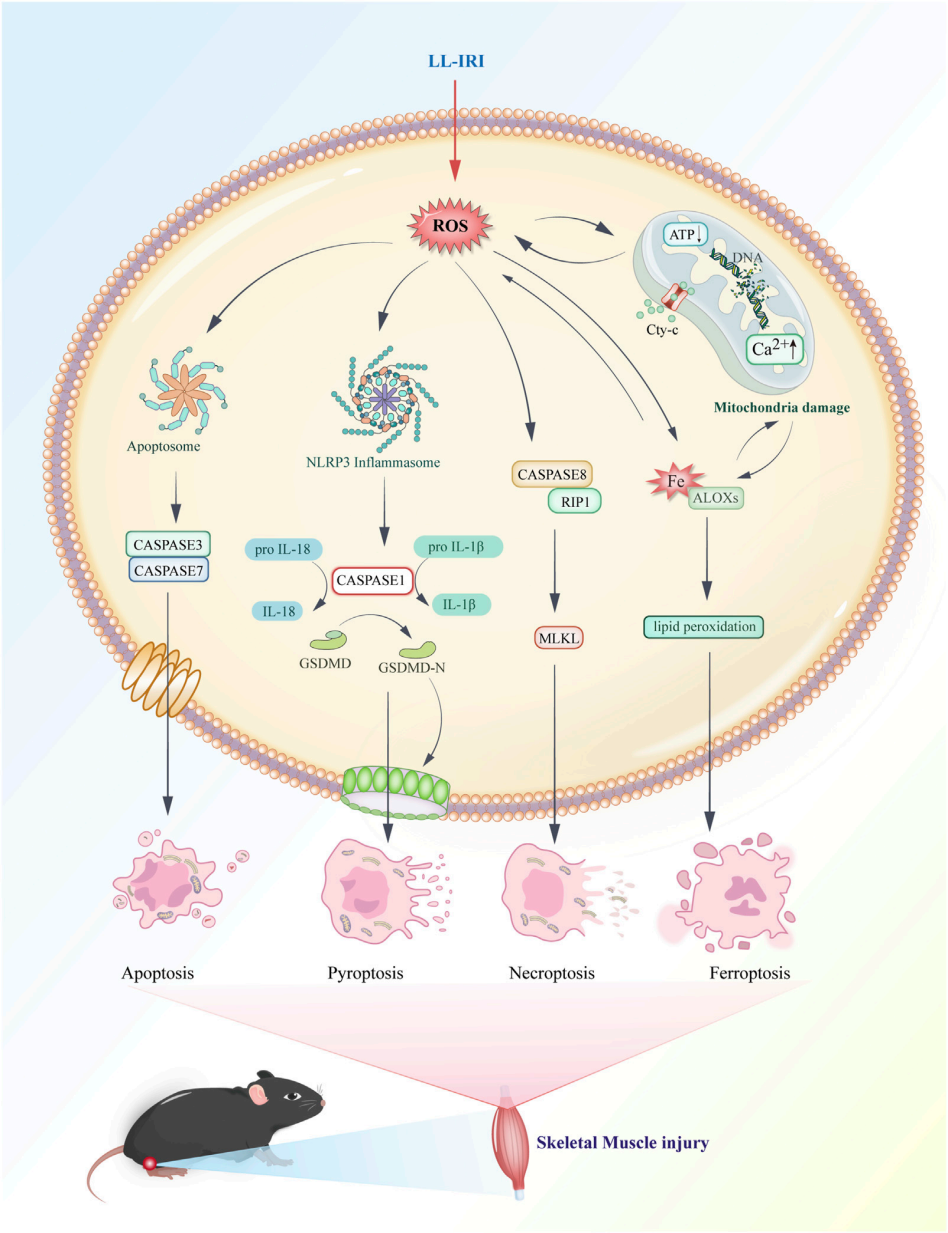
Major mechanisms of cell death in lower-limb ischemia–reperfusion injury.

Ferroptosis, necroptosis, and pyroptosis release pro-inflammatory signals in the microenvironment. Conversely, apoptosis is considered “silent,” and it suppresses subsequent immune responses (Bertheloot et al., 2021). Necroptosis, also known as programmed necrosis, occurs in pathological conditions such as IRI. It is triggered by cellular stress or the activation of death receptors such as TNF receptor-1 and Fas receptor (Wang and Kanneganti, 2021). There is an association between necroptosis and inflammation in the pathogenesis of IRI. Apoptosis is a form of programmed cell death characterized by cell and nuclear shrinkage while maintaining membrane integrity (Li et al., 2020). Compared with necrosis, apoptosis is less immunogenic, and it is executed via both intrinsic and extrinsic pathways. Specifically, apoptosis is executed by activation of effector caspases such as CASP3 and CASP7, occurring downstream of initiator caspases CASP8, CASP9, and CASP10. Pyroptosis is driven by membrane pores formed by activated members of the gasdermin family, with gasdermin D being the prototype activated by inflammatory caspases (CASP1 in mice or CASP4/5 in humans). Necroptosis is achieved through the formation of mixed lineage kinase domain-like pseudokinase pores, which occur after phosphorylation of targets downstream of the receptor-interacting protein kinase 1 (RIPK1) and RIPK3 signaling axis. Ferroptosis is a novel form of programmed cell death closely related to cellular dependence on iron (Jiang et al., 2021). It plays a significant regulatory role in various diseases such as IRI, cancer, neurological disorders, and acute kidney injury. Its characteristics include the accumulation of lipid ROS. Unlike the typical necrotic morphology involving cell and organelle swelling and cell membrane rupture, ferroptosis also lacks traditional features of apoptosis, such as cell shrinkage, chromatin condensation, formation of apoptotic bodies, and breakdown of the cellular cytoskeleton. In contrast to autophagy, ferroptosis does not form typical enclosed double-membrane structures (autophagosomes). Morphologically, nuclear alterations in ferroptosis primarily manifest as significant mitochondrial shrinkage, increased membrane density, and reduced or lost mitochondrial cristae, marking ferroptosis as a distinct form of cell death (Yan et al., 2021). The cell death mechanisms involved in lower limb ischemia-reperfusion, as described above, are illustrated in Figure 3.

## Progress in LL-IRI–related treatments

Recent studies indicate that the development of LL-IRI involves a cascade of inflammatory processes, including neutrophil infiltration, endothelial cell damage, excessive cytokine release, and ROS generation (Kan et al., 2018). Currently, oxidative stress is recognized as a central mechanism in the pathogenesis of this condition, with ROS playing a crucial role. Eukaryotic cells have intricate systems to regulate ROS production and responses (Cheung and Vousden, 2022). ROS are primarily produced by neutrophils, myocytes, vascular endothelial cells (VECs), and perivascular tissues. In cellular signaling, ROS act as key mediators and regulators, influencing the activity of kinases, phosphatases, transcription factors, and cytoskeletal proteins. They are involved in various physiological processes, including pathogen defenses, angiogenesis, and response to fibrotic stimulus-induced injury.

Furthermore, ROS contribute to pathophysiological processes such as endothelial dysfunction, vascular inflammation, and mitochondrial myopathy (Bottoni et al., 2022). Normally, a delicate balance is maintained between the generation and elimination of ROS (Zhang et al., 2016). However, when ROS production exceeds the body’s ability to clear them, oxidative stress ensues. This imbalance leads to peroxidation and oxidative damage in cellular components across various tissues, ultimately resulting in tissue dysfunction.

Redox signaling is particularly critical in ischemia-reperfusion, as it is regulated by redox reactions (Fukai and Ushio-Fukai, 2020). During ischemia, aerobic respiration is suppressed, and hypoxia drives anaerobic respiration, reducing oxidative reactions and increasing lactate release. Upon reperfusion, a sudden influx of oxygen into the tissue leads to the excessive production of ROS. This ROS surge causes oxidation of cellular components and initiates a cascade of inflammatory responses, leading to tissue damage. Thus, targeting oxidative stress is crucial for treating LL-IRI. Additionally, strategies to mitigate mitochondrial damage, protect endothelial cells, promote angiogenesis, and modulate anti-inflammatory responses and apoptosis are also important for alleviating LL-IRI.

### Non-pharmacological treatment strategies for LL-IRI

Extensive research has been dedicated to the prevention and treatment of LL-IRI. In vascular surgery, ischemic preconditioning and postconditioning have been established as effective methods with promising applications for protecting organs and muscles.

Remote ischemic preconditioning (RIPC) is a therapeutic intervention that involves brief, non-lethal ischemia in remote organs to activate cellular and neural pathways. These pathways target mitochondria, prompting the opening of ATP-dependent mitochondrial potassium channels and inhibiting the mPTP. RIPC was reported to reduce ROS production and protect organs during IRI (Aboo Bakkar et al., 2018; Sprick et al., 2019). Clinical studies demonstrated that RIPC can prevent mitochondrial fusion, thereby preventing LL-IRI and improving muscle mitochondrial function. Additionally, RIPC protects skeletal muscle by eliminating acetaldehyde and free radicals (Mansour et al., 2012; Leurcharusmee et al., 2022). RIPC has also been found to reduce kidney injury caused by LL-IRI by decreasing the shear rate in the brachial artery and increasing flow-mediated dilation, thereby alleviating local endothelial dysfunction. Studies recorded significant reductions in serum creatinine, urea, cystatin C, and β-2 microglobulin levels; urine creatinine levels; and the glomerular filtration rate following RIPC (Kasepalu et al., 2020). Different RIPC protocols involving varying cycles and durations of ischemia and reperfusion can lead to significantly different postoperative outcomes; thus, the timing of RIPC should be carefully considered preoperatively (Leurcharusmee et al., 2022).

In addition to RIPC, endovascular shunting (ES) and intraoperative hemodialysis are also used for patients with LL-IRI. ES is a well-established technique used to reduce the ischemic time following acute arterial occlusion or prevent inadequate perfusion after complex open vascular or endovascular surgeries. ES involves connecting two sheaths with catheters, including one proximal and one distal to the arterial occlusion. Clinically, the use of ES in acute limb ischemia surgeries did not lead to significant clinical reperfusion injury postoperatively. However, ES is limited by its flow capacity, necessitating caution when shunting to tissues with high oxygen demands (Österberg et al., 2014).

Clinical studies demonstrated that intraoperative blood drainage using hemodialysis during thrombectomy can restore normal circulation to severely ischemic lower limbs, beneficially affecting potassium levels, acidic metabolites, and oxygen free radicals during reperfusion stabilization (Mutirangura et al., 2009). Nevertheless, this therapy is expensive, and it demands significant economic resources from patients.

Low-intensity laser therapy has also been revealed to reduce IRI in rat skeletal muscles by inducing the synthesis of antioxidants and other protective cellular proteins (Takhtfooladi et al., 2015). In addition, hypothermia therapy can mitigate LL-IRI–induced muscle damage and decrease post-reperfusion compartment pressure (Simon et al., 2018).

### Drug treatment strategies for LL-IRI

**FIGURE 4:**
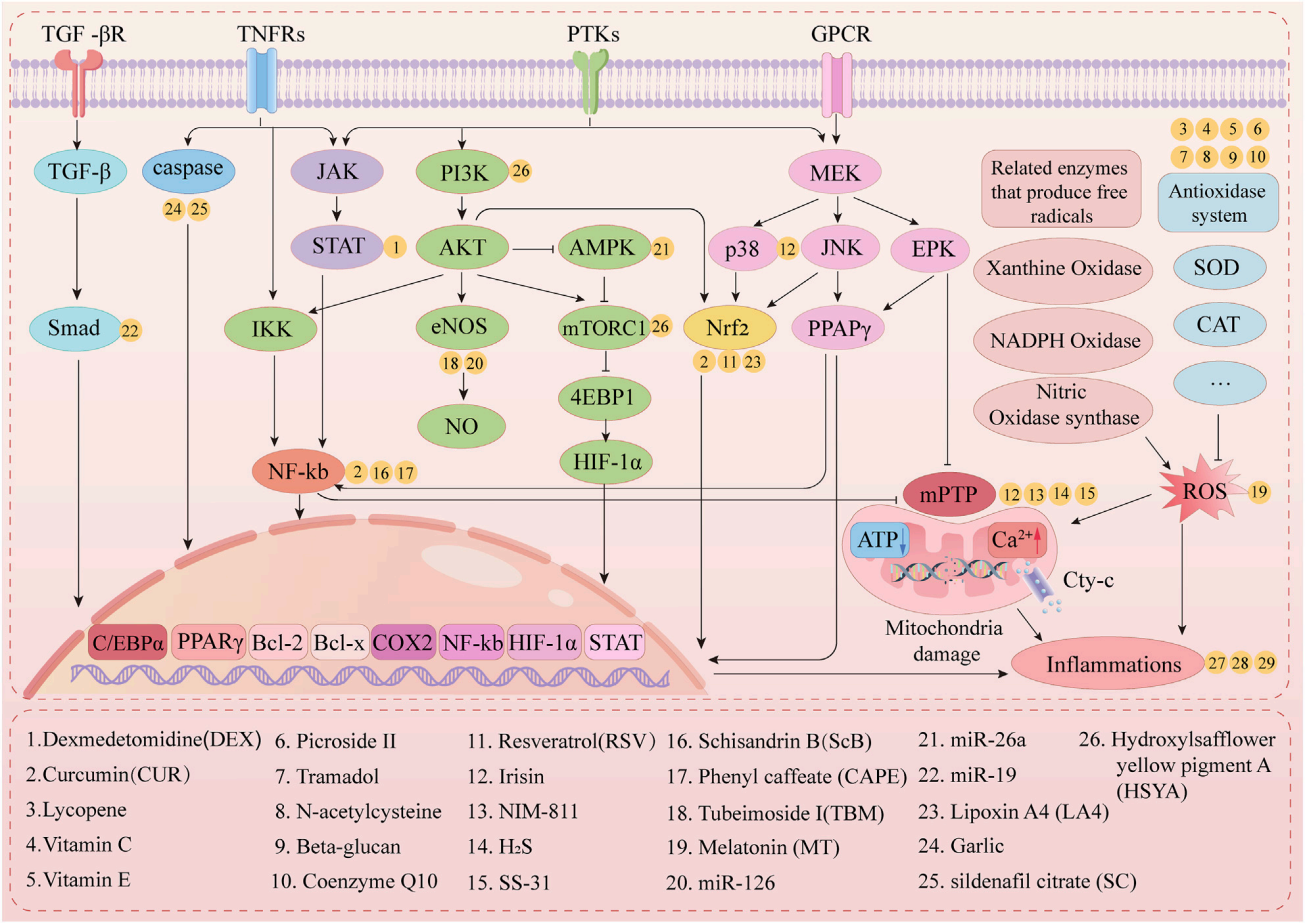
Mechanistic pathway of lower-limb ischemia–reperfusion drug therapy.

As presented in Figure 4, multiple drugs alleviate LL-IRI through various mechanisms.

#### Antioxidant stress response

Numerous pathological mechanisms are responsible for LL-IRI, with inflammation and oxidative stress playing key roles. Organs affected by IRI exhibit increased ROS levels, which are correlated with reduced expression of antioxidant proteins. Furthermore, damage induced by excessive ROS can lead to the release of pro-inflammatory cytokines. Thus, inhibiting the production and release of ROS and inflammatory cytokines is considered a viable strategy for mitigating LL-IRI.

Nuclear factor erythroid 2-related factor 2 (NRF2) is a transcription factor recognized as a major regulator of oxidative stress and metabolic homeostasis (Dodson et al., 2019; Cen et al., 2022). The dysregulation of NRF2 signaling is associated with many oxidative stress-related conditions (Tonelli et al., 2018). NRF2 is involved in regulating programmed functions in response to oxidative stimuli, such as autophagy, inflammasome assembly, endoplasmic reticulum stress/unfolded protein response, mitochondrial biogenesis, and stem cell regulation. NRF2 mediates cellular oxidative levels and oxidant signaling by regulating the expression of three groups of antioxidant response element-dependent genes: drug metabolizing enzymes/transporters, antioxidant enzymes/proteins, and oxidant signal proteins (Ma, 2013). NRF2 activators have been implicated as potential drugs for increasing antioxidant capacity and alleviating pathology. Induction of antioxidant enzymes, particularly through NRF2, is a principal approach in developing antioxidant therapies (Forman and Zhang, 2021). This antioxidative property contributes to the treatment of LL-IRI.

Resveratrol (RSV) is a phenolic compound first isolated from the roots of *Veratrum grandiforum* O. Loes, a plant of the *Melanthiaceae* family. Several studies described its ability to extend the lifespan of various model organisms (Clarke and Mostoslavsky, 2020). Song et al. confirmed the ability of RSV to block Nrf2 signal transduction, revealing that RSV is protective against IRI and oxidative stress. Experiments suggested that RSV can mitigate LL-IRI–induced vascular endothelial injury by modulating Keap1/Nrf2-mediated oxidative stress (Song et al., 2021).

Lipoxin A4 (LXA4) is a biologically active metabolite of arachidonic acid with anti-inflammatory properties that is capable of inhibiting neutrophil infiltration and reducing ROS generation (Gonçalves et al., 2023). Recent studies revealed that LXA4 can protect multiple organs from IRI and significantly improve histological damage scores in IRI-injured muscle tissue (Zong et al., 2017). The mechanism of action of LXA4 might involve activation of the Nrf2/HO-1 signaling pathway. LXA4 holds potential as a promising therapeutic agent for muscle tissue IRI (Peng et al., 2022).

Curcumin (CUR) and dexmedetomidine (DEX) have been demonstrated to protect against IRI in various organs. CUR has a long usage history in Chinese medicine, and it possesses diverse pharmacological properties, including antioxidant, anti-inflammatory, anti-viral, anti-microbial, anti-fungal, and anti-cancer activities. Recent data illustrated that CUR can increase Hsp70 expression and antioxidant enzyme activity, thus inhibiting ROS generation (Giordano and Tommonaro, 2019). DEX is a highly selective α2-adrenergic agonist, and it is used as a sedative, anxiolytic, and analgesic agent (Cheng W. et al., 2019). The protective effects of DEX might involve inhibition of the JAK2/STAT3 signaling pathway, which reduces the amount of ROS in the body (Cai S. et al., 2022). CUR has been found to mitigate organ damage in various IRI models, such as that in the lungs, kidneys, liver, heart, and ovaries, possibly through the Nrf2 pathway. Intraperitoneal administration of CUR and/or DEX reduced hind limb IRI in rats; however, further large-scale studies are needed to validate their potential benefits (Karahan et al., 2016).

Lycopene is a potent antioxidant carotenoid that is widely present in red fruits and vegetables (Wu et al., 2022). It exhibits protective effects against hindlimb muscle IRI in rats through enhancing the activity of antioxidant enzymes such as SOD, glutathione peroxidase (GPX), and catalase (CAT); reducing malondialdehyde (MDA) levels; and significantly attenuating the inflammatory response following LL-IRI (Kirişçi et al., 2020).

Vitamins C and E are potent antioxidants that exhibit synergistic effects when used together. Their antioxidant actions involve rapid reactions with superoxide anion (O_2_^−^), peroxyl radical (HOO^−^), and hydroxyl radical (OH^−^), leading to the formation of semidehydroascorbic acid. Semidehydroascorbic acid can further react with NADH to generate active ascorbate, thereby continuing the function of scavenging free radicals. Animal experimental studies illustrated that vitamins C and E can mitigate oxidative stress and tissue inflammation associated with LL-IRI. They achieve this by reducing the levels of MDA and reactive oxygen intermediates and increasing the activities of SOD and GPX (Arató et al., 2010; Kim et al., 2017).

Baicalein II is a glycoside derivative known for its various pharmacological effects, including antioxidant, anti-apoptotic, anti-inflammatory, anti-cancer, neuroprotective, hepatoprotective, cholesterol-lowering, and immunomodulatory activities (Zhou et al., 2023). Experimental studies demonstrated that baicalein II can enhance endogenous antioxidant enzyme activity, thereby scavenging excessive oxygen free radicals and protecting against LL-IRI (Kılıç et al., 2017).

Tramadol hydrochloride is an effective analgesic for acute and chronic pain, such as cancer pain, neuropathic pain, and postoperative pain (Şen et al., 2020). Tramadol has been demonstrated to alleviate IRI in heart or brain tissue (Akkurt et al., 2018). Tramadol exerts antioxidant effects by reducing lipid peroxidation levels and enhancing antioxidant enzyme activity and displays potential therapeutic benefits in treating LL-IRI (Takhtfooladi et al., 2014).

Acetylcysteine, β-glucan, and coenzyme Q10 have antioxidant and anti-inflammatory effects against reperfusion injury (Testai et al., 2021; Croome, 2023). In mouse models of hind limb IRI, administration of any of these antioxidant agents resulted in decreased levels of MDA and significantly increased levels of GPX and SOD, and mouse survival rates were generally increased. These results suggest that N-acetylcysteine, β-glucan, and coenzyme Q10 contribute to reducing damage from LL-IRI (Bolcal et al., 2007).

#### Mitigating mitochondrial damage

Mitochondrial dysfunction plays a crucial role in the development of LL-IRI. Key aspects include impaired mitochondrial oxidative capacity and premature mPTP opening. An imbalance between ROS production and clearance because of mitochondrial dysfunction, along with increased inflammation, exacerbates oxidative stress. Mitochondria are both participants in ischemia-reperfusion and the targets of its effects (Zeng et al., 2023).

mPTP opening plays a crucial role in the development of IRI, contributing to cell death. Irisin, a 112-amino acid hormone primarily secreted in skeletal and cardiac muscle, exhibits various pharmacological effects including antioxidant, anti-apoptotic, anti-inflammatory, neuroprotective, and hepatoprotective properties. In the context of ischemic heart injury, irisin significantly improved post-ischemic ventricular function and reduced infarct size by inhibiting mPTP opening, preventing mitochondrial swelling, and protecting mitochondrial function (Wang et al., 2017). Recent studies confirmed that irisin plays an important role in mitigating LL-IRI by improving mitochondrial function, simultaneously reducing oxidative stress levels and inflammatory responses (Küçük et al., 2021).

N-methyl-4-isoleucine-cyclosporin (NIM-811) is a mitochondria-specific drug that prevents LL-IRI by inhibiting the opening of the PTP (Garbaisz et al., 2014).

Hydrogen sulfide (H_2_S) is a gasotransmitter that, along with nitric oxide (NO) and carbon monoxide, initiates various signaling pathways within cells. Traditionally known for its characteristic rotten egg odor and toxic properties, recent evidence suggests that trace amounts of H_2_S can affect vascular dilation, metabolism, cell apoptosis, and mitochondrial electron transport chain, among other signaling pathways (Bełtowski, 2015). H_2_S functions by inhibiting the opening of the mPTP, thereby mitigating mitochondria-induced cell death. This effectively protects limbs from ischemia-reperfusion-induced mitochondrial damage, preserves mitochondrial energy metabolism, and prevents excessive ATP consumption (Fu et al., 2019).

During the process of IRI, increased ROS levels cause mitochondrial swelling, dysfunction, and even rupture, leading to the release of cytochrome c from mitochondria. Studies revealed that cytochrome c leakage from mitochondria initiates apoptotic signaling cascades, ultimately resulting in cell death (Chen et al., 2006). SS-31 (elamipretide), a mitochondria-targeted antioxidant, has been reported to improve mitochondrial function, and its efficacy against various diseases was reported in clinical trials (Zhang et al., 2022). SS-31 interacts with mitochondrial cardiolipin, reducing mitochondrial ROS production, enhancing ATP production, preventing mitochondrial swelling, clearing ROS, and reducing oxidative stress (Hao et al., 2015). Experimental studies demonstrated the preventive and therapeutic effects of SS-31 in a mouse model of LL-IRI (Cai et al., 2018).

#### Anti-inflammatory response

Inflammation is a local reaction in various diseases characterized by neutrophil infiltration. Thrombin participates in the multiple processes of inflammatory cascade reactions, and thus, coagulase inhibitors might be useful for IRI treatment because they reduce the neutrophil–endothelial interaction mediated by coagulase (Iba et al., 2024). Many experiments found that antithrombin III preconditioning can reduce inflammatory factor levels and alleviate IRI in the heart and kidneys (Huang et al., 2019; Firdus et al., 2022). Low-molecular-weight heparin (LMWH) is an antithrombotic drug that depends on AT-III. In addition to its anti-coagulant and anti-thrombotic effects, LMWH can attenuate inflammatory responses and regulate calcium homeostasis. Jin et al. demonstrated that administering LMWH after limb surgery mitigated IRI and reduced inflammation in rat hind limbs caused by tourniquet application (He et al., 2021).

C1 esterase inhibitor (C1 INH) is a major regulator of the complement system, as it interacts with all three pathways of complement activation and plays a crucial role in both coagulation and the kinin system. C1 INH acts on various inflammatory cascades related to IRI. By inhibiting kallikrein release, FXIa, FXIIa, and the complement system, C1 INH modulates inflammation and thrombus formation processes associated with IRI. Experimental evidence suggests that C1 INH treatment significantly reduces the levels of pro-inflammatory cytokines such as TNF-α, reduces skeletal muscle edema, and preserves muscle cell viability to protect against peripheral IRI. Therefore, C1 INH represents a promising therapeutic option that can reduce complex and prolonged IRI requiring tourniquet application in surgical procedures involving the lower limbs (Duehrkop et al., 2013).

Neutrophils play a crucial role in ischemia–reperfusion-induced skeletal muscle injury. Microtubules are necessary for neutrophil activation under various stimuli. Studies reported that the microtubule blocker colchicine can significantly reduce ischemia–reperfusion-induced skeletal muscle injury and edema in rats by reducing the release of inflammatory factors such as TNF-α and IL-1β (Orhan et al., 2024).

NF-κB plays a major role in the pathogenesis of skeletal muscle IRI. Schisandrin B (ScB), the most abundant lignan in the plant *Schisandra chinensis*, inhibits oxidative stress and inflammatory responses induced by hind limb IRI in rats via the MAPK/NF-κB pathway, suggesting that ScB can mitigate hind limb IRI-induced tissue damage (Zhu et al., 2017). Furthermore, caffeic acid phenethyl ester, a potent NF-κB inhibitor, protected rat skeletal muscle from IRI by suppressing the NF-κB signaling pathway and reducing tissue inflammation (Andrade-Silva et al., 2009).

#### Protection of VECs and promotion of angiogenesis

Microvascular dysfunction is a main pathogenic factor of IRI. Microvascular stress during ischemia-reperfusion can be exacerbated by impaired vascular function, reduced capillary perfusion, leuco-endothelial cell adhesion, albumin leakage, and interstitial edema. However, the molecular mechanisms of microvascular injury and endothelial cell death remain unclear. Alleviating endothelial cell death might represent a promising therapeutic strategy for the management of ischemia–reperfusion-related diseases (Chen et al., 2023).

Saffron is widely used in traditional Chinese medicine to treat cardiovascular and cerebrovascular diseases. Hydroxysafflor yellow A (HSYA), the main active component of saffron, exhibits multiple biological activities. HSYA is used in the treatment of myocardial and cerebral ischemia, hypertension, atherosclerosis, vascular dementia and traumatic brain injury (Bai et al., 2020). In a mouse hind limb ischemia model, HSYA enhanced blood flow recovery and increased capillary and small artery density. This suggests that HSYA promotes angiogenesis, indicating its significant potential efficacy in treating LL-IRI (Chen et al., 2016).

Tubeimoside I (TBM) is an extract from the traditional Chinese herb Tubeimu, and it has proven efficacy as an anti-tumor agent against various human cancers (Yang et al., 2021b). In traditional Chinese medicine, TBM has been used for more than 1000 years to treat acute mastitis, snake bites, inflammatory diseases, and tumors, in addition to uses as a detoxifying agent (Yang et al., 2021a).

Melatonin (MLT) is a hormone secreted primarily in the pineal gland, and it is involved in regulating neovascularization and inhibiting tumor development. Recent studies found that MLT can effectively combat oxidative stress injury in vascular endothelial cells, protect vascular endothelial cells by reducing ROS, promote neovascularization, and thus protect the myocardium from the effects of IRI (Cheng J. et al., 2019; Mao et al., 2020). Therefore, MLT might be useful in the treatment of LL-IRI.

MicroRNAs (miRNAs) comprise a class of small RNAs of approximately 21–25 nucleotides in length. They are widely present in eukaryotes, and their dysregulation is closely associated with the occurrence and progression of various diseases (Wu et al., 2014). miRNAs can regulate both IRI and ischemic preconditioning and postconditioning. miR-126 is the most highly expressed miRNA in endothelial cells, including those in the heart, lungs, and other organs (Qin et al., 2013). miR-126 is highly enriched in endothelial cells, and it plays a regulatory role in vascular integrity and pathophysiology. In particular, it activates the PI3K/Akt/eNOS signaling pathway; promotes the production of SOD, NO, and vascular endothelial growth factor; and enhances cell proliferation and lumen formation capabilities while inhibiting ROS levels and the expression of IL-6, IL-10, and TNF-α, thereby attenuating apoptosis and protecting endothelial cells against IRI and inflammation (Yang et al., 2017). Endothelial dysfunction in VECs plays a crucial role in IRI-related diseases, with miRNAs being key factors in this process. In a rat model of IRI, downregulation of miR-26a and concomitant upregulation of PFKFB3 were observed in vascular tissues (Hanson et al., 2023). Wu et al. reported that miR-26a RNA mitigates VEC damage induced by LL-IRI in rats by inhibiting PFKFB3 and activating the AMPK pathway (Wu et al., 2019). Krüppel-like factors (KLFs) are major players in transcriptional networks that control proliferation, apoptosis, differentiation, development, and tumorigenesis. As a transcriptional regulator of the KLF family, KLF10 has been identified as a target gene regulating the expression of various genes and signaling pathways, and it is also a target gene of miR-19 (Yuce and Ozkan, 2024). In the vascular epithelial tissues of rats with LL-IRI, miR-19 overexpression reduced the expression of KLF10, TGF-β1, and Smad2/3. Reduced miR-19 expression inhibits VEC proliferation, arrests VECs in the G1 phase, and induces apoptosis of these cells following LL-IRI (Xu et al., 2018). These findings suggest that miR-19 acts as an inhibitor of IRI-induced VEC damage by inhibiting KLF10 via the TGF-β1/Smad signaling pathway.

#### Inhibition of cell apoptosis

Repeated ischemia-reperfusion therapy performed on organs or tissues after ischemia following LL-IRI significantly reduces apoptosis levels in vascular tissues, decreases the expression of p-p38 MAPK and its downstream factor ATF-2, and alleviates vascular damage in the lower-limb arteries. Co-administration of MAPK pathway inhibitors during post-treatment after LL-IRI further reduces the rate of apoptosis. Inhibiting the MAPK pathway additionally suppresses apoptosis, facilitating recovery from LL-IRI (Guan et al., 2018).

Evidence indicates that the Akt signaling pathway plays a significant role in cardiomyocyte apoptosis and myocardial IRI (Yu et al., 2015). Pei et al. observed in a mouse model of myocardial IRI that activation of the Akt signaling pathway can reduce the protein expression of Bax, thereby significantly protecting tissues from the effects of IRI (Pei et al., 2016). In a model of LL-IRI established by Chen et al., silencing the *PDCD4* gene activated the Akt signaling pathway, alleviating VEC damage caused by LL-IRI in rats. PDCD4 might thus be an important therapeutic target for LL-IRI (Chen et al., 2019). Silencing of the long non-coding RNA (lncRNA) antisense RNA 1 can further inhibit oxidative stress, exert anti-apoptotic effects, reduce inflammatory responses, and protect against acute LL-IRI in rats by activating the PI3K/Akt pathway (Shen et al., 2020).

Acute IRI led to reduced systolic tone, morphological damage, macrophage infiltration, elevated TNF-α levels, and apoptosis in gastrocnemius muscle. Chronic intermittent hypoxic depression (CIHH) refers to high-altitude exposure over 6 months, with at least 30% spent alternating shifts between high and low altitudes (Tian et al., 2021). In a skeletal muscle IRI mouse model, CIHH pretreatment (5,000 m altitude, 6 h/day for 28 days) improved systolic function and reduced apoptosis, macrophage infiltration (CD68^+^) and TNF-α levels while mitigating inflammation (Cheng W. J. et al., 2019).

Garlic, traditionally used in medicine, has demonstrated protective effects against IRI in various organs, including the heart, brain, kidneys, and liver (Czompa et al., 2018; Gomez et al., 2019; Lasheen et al., 2019; Asdaq et al., 2021; Li et al., 2022). Garlic treatment resulted in significant decreases in caspase-3 (CASP3) expression, apoptosis, myofibrillar degeneration, inflammatory infiltration, and skeletal muscle edema were significantly reduced (Abd El-Mottaleb et al., 2019).

Sildenafil citrate, a phosphodiesterase-5 inhibitor, delays cGMP and NO degradation (Zaobornyj et al., 2019). Early application of sildenafil citrate reduced the apoptosis rate of skeletal muscle cells by decreasing caspase-3 expression in a rat model of hind limb ischemia, reducing skeletal muscle cell damage caused by IRI and thus protecting against skeletal muscle LL-IRI–related damage (Armstrong et al., 2013).

## Conclusion

The exact mechanisms underlying IRI in clinical settings remain unclear, hindering the development of effective treatments. A thorough understanding of its complex pathophysiology and cellular death pathways is crucial for discovering novel therapeutic approaches.

Historically, cell death pathways were considered discrete and non-interacting. However, emerging evidence has revealed intricate crosstalk and regulatory relationships among apoptosis, pyroptosis, necroptosis, and ferroptosis. The selection of these death modalities is predominantly dictated by the extent of tissue damage and microenvironmental conditions. The pathogenesis of IRI exhibits remarkable complexity across multiple dimensions. The orchestration of cell death has been recognized as a pivotal mechanism to mitigate IRI, with distinct cell death modalities exerting critical yet heterogeneous roles that warrant systematic investigation. Current therapeutic strategies predominantly target conventional apoptotic pathways, overlooking alternative death mechanisms. Furthermore, although the molecular intricacies of IRI require further elucidation, most interventions remain confined to isolated signaling pathways and continue to focus on apoptotic pathways, neglecting other cell death modalities. A comprehensive understanding of the pathophysiology of IRI will inform the development of multimodal therapeutic strategies, thereby significantly enhancing patient survival rates and improving quality of life through the precision modulation of cell death networks.
